# Human Physiology

**Published:** 1844-10

**Authors:** 


Art. VI.—*Human Physiology; with upwards of Three Hundred Illustrations.* By Robley Dunglison, m.d., Professor of the Institutes of Medicine in Jefferson Medical College, &c. &c. Fifth Edition, greatly modified and improved.—*Philadelphia,* 1844. In Two Vols. pp. 1304.

We have, on two former occasions, brought this excellent work under the notice of our readers, and we have only now to say that, instead of falling behind in the rapid march of physiological science, each succeeding edition brings it nearer to the van. Vithout increasing the bulk of the treatise, the author has contrived to introduce a large quantity of new matter into this edition from the works of Valentin, Bischoff, Henle, Wildbrand, Müller, Wagner, Mandl, Gerber, Liebig, Carpenter, and Todd and Bowman, as well as from various monographs which have appeared in the Cyclopædias, transactions of learned societies, and journals. To make room for this, a great proportion of obsolete matter, which we always considered a blemish in the work, has been removed, and there is now very little that we should care to see omitted. To those who desire a work on physiology more extended than that of Dr. Carpenter, and more readable than that of Professor Müller, we can strongly recommend this treatise of Dr. Dunglison's. The large mass of references which it contains, renders it a most valuable bibliographical record, and bears the highest testimony to the zeal and industry of the author. There is only one department in which we think his work deficient; and that is in regard to subjects of microscopical inquiry. From various hints let fall by him, we infer that he has not been in the habit of using the microscope, in its present improved form at least, and that he is consequently unable to test and verify the observations in which the last few years have been so fertile. Hence we find him expressing doubt on some points which, we think, have been satisfactorily determined; and speaking confidently as to others, on which we should have felt more hesitation.

To the present edition have been added ninety new wood-engravings; most of them being those prepared (by the same publisher) for the illustration of an edition of Dr. Carpenter's Human Physiology, which was reprinted in Philadelphia last year. We are happy to see that, owing to the great demand which at present exists in America for physiological works, there is ample room for both these treatises.

